# The second beat matters: coupling intervals and sudden cardiac death risk in hypertrophic cardiomyopathy

**DOI:** 10.1093/europace/euag246

**Published:** 2026-09-17

**Authors:** Rasmus Bork Dinesen, Vincent Probst, Jacob Tfelt-Hansen

**Affiliations:** Department of Cardiology, Copenhagen University Hospital, Rigshospitalet, Blegdamsvej 9, 2100 Copenhagen, Denmark; Department of Cardiology, Inserm UMR 1087, CIC INSERM, Institut du Thorax, CHU Nantes, Nantes, Boulevard Jacques-Monod, 44093 Nantes Cedex 1, France; Department of Cardiology, Copenhagen University Hospital, Rigshospitalet, Blegdamsvej 9, 2100 Copenhagen, Denmark; Department of Forensic Medicine, Copenhagen University, Frederik V's Vej, 2100 Copenhagen, Denmark

**This editorial refers to ‘Associations between specific non-sustained ventricular tachycardia characteristics and sudden cardiac death in hypertrophic cardiomyopathy’ by Y. Zhang *et al*., https://doi.org/10.1093/europace/euag034.**

Predicting the future is a challenging task, yet it is an inherent part of a clinician’s daily practice. Few clinical challenges are more difficult and more consequential than estimating the risk of sudden cardiac death (SCD) for primary prevention. In patients with hypertrophic cardiomyopathy (HCM), this challenge assumes particular importance, given that SCD often represents the first and only manifestation of the disease.^1^ Accordingly, identifying patients at increased risk of SCD is a critical task, as it directly impacts decisions regarding primary prevention with an implantable cardioverter defibrillator (ICD).^2^ Risk stratification tools, such as the HCM Risk-SCD model endorsed by the European Society of Cardiology, have therefore become central to the contemporary management of patients with HCM.^3^ The implementation of such models, alongside advancement in medical and device therapy, has contributed to a global decline in SCD rates in patients with HCM.^4^ Nevertheless, further refinement of risk assessment is required to improve precision with which we identify high-risk patients with HCM who may benefit of an ICD, while equally ensuring avoidance of unnecessary device implantation to minimize ICD-related complications, especially in those with an intermediate risk.^5^

In this issue of *Europace*, Zhang *et al*. aim to provide such refinement, by extending the prognostic understanding of non-sustained ventricular tachycardia (NSVT) in relation to SCD prediction in patients with HCM.^6^ Non-sustained ventricular tachycardia has long served as a well-documented risk marker, incorporated as a dichotomous variable in both the ESC and ACC/AHA risk tools.^3,7^ However, its value as a binary predictor has been questioned—contemporary studies have suggested that certain characteristic traits such as rapid rate and longer duration are associated with increased SCD risk, though the prognostic value of these characteristics remains debatable.^8,9^ Moving beyond these characteristics, Zhang *et al*. examine whether the coupling intervals of NSVT carry independent predictive value for SCD risk—and whether their measurement can distinguish the truly malignant from the prognostically benign.

Designed as a retrospective multicentre study, Zhang *et al*. enrolled 2584 patients with HCM, all of whom underwent 24-h Holter monitoring within 2 days of inclusion. Based on Holter findings, patients were categorized into two groups: those with NSVT (*n* = 320, 12.4%) and those without. Among those with NSVT, multiple coupling intervals were systematically measured and adjudicated by an experienced electrophysiologist. Patients with NSVT were subsequently further subdivided into two groups, those who experienced SCD and those who did not, and had their coupling intervals compared. The second coupling interval—defined as the RR-interval between the first and second beat of the NSVT episode—was significantly shorter in the SCD group. Return of Spontaneous Circulation (ROC) curve analysis confirmed the second coupling interval as the strongest prognostic discriminator among all NSVT characteristics examined, with a C-statistic of 0.763 (95% CI: 0.645–0.881), and identified an optimal cut-off of 394 ms (sensitivity 0.706; specificity 0.805).

The large multicentre cohort and systematic electrophysiologist-confirmed coupling interval measurements lend credibility to the identified cut-off. However, the use of a single 24-h Holter recording to assess NSVT is a notable limitation—prior studies have shown that 24-h recordings miss more than half of all NSVT episodes in patients with HCM, raising the question of whether the coupling intervals captured are truly representative of each patient’s arrhythmic profile.^10^

Applying the cut-off threshold, Zhang *et al*. stratified the entire cohort into three prognostic groups: patients without NSVT (*n* = 2264), those with NSVT and a long second coupling interval (≥394 ms, *n* = 249), and those with NSVT and a short second coupling interval (<394 ms, *n* = 71). Over a median follow-up of 4.4 years, 64 patients (2.5%) experienced SCD. In both univariable and multivariable Cox regression analyses, patients with a short second coupling interval carried a markedly elevated SCD risk compared with those without NSVT [multivariable hazard ratio (HR) 8.36, 95% confidence interval (CI) 4.33–16.12, *P* < 0.001] (see Table 1). Interestingly, patients with a long second coupling interval carried an SCD risk statistically indistinguishable from those without NSVT—suggesting that the conventional dichotomous NSVT definition conflates two prognostically distinct populations, only one of which warrants the designation ‘malignant’.

**Table 1:** Prognostic value of fast and slow NSVT in patients with hypertrophic cardiomyopathy

Model	Sudden cardiac death	
Univariable model	HR (95% CI)	*P*-value
Fast NSVT^a^	9.83 (5.20–18.56)	< 0.001
Slow NSVT^b^	1.02 (0.41–2.56)	0.968
Multivariable model I		
Fast NSVT	10.79 (5.66–20.54)	<0.001
Slow NSVT	1.14 (0.45–2.87)	0.789
Multivariable model II		
Fast NSVT	8.36 (4.33–16.12)	<0.001
Slow NSVT	0.79 (0.31–2.03)	0.623

Cox proportional hazard models estimating the prognostic value of the second coupling of NSVT for predicting sudden cardiac death using the no NSVT group as reference.

CI, confidence interval; HR, hazard ratio; NSVT, non-sustained ventricular tachycardia.

^a^Fast NSVT: short second coupling (<394 ms).

^b^Slow NSVT: long second coupling (>394 ms).

The identification of the second coupling interval as a prognostic discriminator represents a genuinely novel contribution to the field of SCD risk stratification in HCM. Its prognostic relevance finds support in the work of Kim *et al*., who studied patients with idiopathic ventricular tachycardia originating from the outflow tract in the absence of structural heart disease.^11^ Strikingly, the second coupling interval was markedly shorter in those with malignant arrhythmias compared with benign ventricular tachycardia, while the first coupling interval did not differ between groups. Zhang *et al*. now extend this observation to the structurally abnormal myocardium of HCM, with a notably similar pattern: it is specifically the second, rather than the first, coupling interval that carries the prognostic signal—suggesting that the accelerating beat-to-beat dynamics within the NSVT episode, rather than its initiation, may be the electrophysiologically relevant feature.

These findings are nonetheless grounded in a relatively small number of events—only 17 of the 320 patients with NSVT experienced SCD during follow-up—which limits the statistical robustness of the coupling interval finding and renders the optimal cut-off susceptible to meaningful change with even small shifts in the data. Additionally, the primary analysis relied on standard Cox regression, which treats non-SCD deaths as censored rather than competing events—an approach that may overestimate the absolute cumulative incidence of SCD when non-arrhythmic mortality is non-negligible. The absence of a competing risks analysis, as employed in other HCM-related studies, is a methodological limitation worth noting.

Beyond its prognostic value as a standalone discriminator, Zhang *et al*. investigate whether incorporating the ‘malignant NSVT’ definition into the ESC-endorsed HCM Risk-SCD model could enhance its performance. They recalibrated the model by replacing the conventional dichotomous NSVT variable with their malignant NSVT definition—restricting the NSVT risk contribution to only patients with a short second coupling interval (<394 ms). The C-statistic of the updated model increased to 0.775 (95% CI: 0.702–0.848), significantly higher than that of the original model refitted to the study cohort at 0.749 (95% CI: 0.674–0.825; *P* = 0.003). Conversely, with respect to calibration, the original model performed marginally better, with a calibration slope closer to the ideal value of 1.0 (1.022, 95% CI: 0.773–2.872) compared with the updated model (1.200, 95% CI: 1.093–2.209)—suggesting that improved discrimination came at a modest cost to calibration accuracy.

The clinical impact of the updated model is nonetheless most apparent in the intermediate-risk grey zone. Of 134 patients with a predicted 5-year SCD risk of 4–6% under the original model, 19 were reclassified as high risk (≥6%)—one of whom experienced SCD—while 25 were reclassified as low risk (<4%), with no SCD events recorded. In a field where intermediate-risk ICD decisions remain clinically challenging, the ability to refine risk stratification at precisely this threshold is where the value of the coupling interval may prove most clinically relevant.

The 394 ms cut-off and the ‘malignant or fast NSVT’ definition were derived and tested within the same single tertiary cohort, and the absence of external validation is an important limitation, particularly given that ROC-derived thresholds are known to be sensitive to the dataset from which they are generated. The observed gain in C-statistic should likewise be interpreted with caution, as model performance evaluated in the derivation cohort tends to overestimate true predictive accuracy in independent populations—a well-recognized phenomenon in cardiovascular risk modelling.^12^ Hence, external validation in independent and ethnically diverse cohorts are an important next step for fast NSVT as a risk marker to change clinical practise and the guidelines.

The study by Zhang *et al*. represents an important and meaningful step forward in the refinement of NSVT as a risk marker of SCD in patients with HCM. The demonstration of the second coupling interval as a novel prognostic discriminator provides important new insights, showing that not all NSVT is equally malignant—and that conventional dichotomous assessment may fail to identify the truly high-risk subgroup. The potential to sharpen the HCM Risk-SCD model at the intermediate-risk threshold, where clinical uncertainty weighs most heavily on ICD decision-making, adds further excitement and relevance to these findings. Looking ahead, a truly comprehensive risk stratification framework for HCM will likely require integration of additional dimensions—including genetic substrate, Cardiovascular magnetic resonance-defined myocardial fibrosis, concomitant atrial fibrillation, socioeconomic determinants of arrhythmic risk, and artificial intelligence-derived electrocardiographic phenotypes—each representing a dimension of risk not yet captured by current stratification models.^13–15^ However, with respect to NSVT specifically, the prognostic value of the second coupling interval shows promising signs. The critical question that remains is whether the 394 ms cut-off will prove robust across different populations and healthcare settings—prospective, multicentre validation will be the decisive test. For now, the coupling interval represents a promising addition to the growing toolkit for SCD risk stratification in HCM, and one that warrants serious attention from both the research community and potentially future guideline committees.
